# Exploring workplace mental health: educator perspectives and factors in the medical education system – a mixed method study

**DOI:** 10.1186/s12909-024-05095-w

**Published:** 2024-02-08

**Authors:** Fatemeh Keshmiri

**Affiliations:** 1 Medical Education Department, Educational Development Center, Shahid Sadoughi University of Medical Sciences, Yazd, Iran; 2 National Agency for Strategic Research in Medical Education, Tehran, Iran

**Keywords:** Psychological health, Workplace mental health, Well-being, Occupational health, Mixed-method study, Faculty member, Educator, Teacher

## Abstract

**Introduction:**

The present study aimed to assess the status of workplace mental health from the viewpoints of educators, and explore their experiences concerning influential factors on occupational mental health at Shahid Sadoughi University of Medical Sciences.

**Methods:**

The study was a sequential mixed-method study that was conducted in quantitative and qualitative phases. In the quantitative phase, the perception of educators (*n* = 205) was assessed by a Workplace Mental Health Questionnaire, including 37 items in 9 categories (including an opportunity to control, an opportunity to use skills, external goals created, environmental diversity, environmental clarity, access to money, physical security, opportunity to contact others, and valuable social status and position). In the qualitative phase, data were collected using semi-structured interviews (*n* = 21) and were analyzed based on the conventional content analysis approach.

**Results:**

The results showed that the status of workplace mental health of educators was at a moderate level (mean (± SD) = 115.87 (±3.21). The highest and lowest scores of the median were reported in the domains of “opportunity for control” (median = 4) and “opportunity to contact others” (median = 2.75), respectively. The theme of “contrast between preferences and disappointments in the development path” with two categories including “induced demotivation of system elements” and “tendencies of promotion” was explored from the educators’ perspective.

**Conclusion:**

The results indicated the moderate level of mental health of educators was influenced by the contrast between preferences and disappointments in the development path. The tendency of educators for promotion was explored as a positive factor in the mental health of educators in the academic environment. A growing desire for creative advancement among educators as a personal factor and a demand to stay updated with all developments as a system factor explained the positive experiences of educators in the university. The results showed the gap between the current situation and the desired state of occupational mental health may result from cultural challenges, lack of adherence to professionalism at the personal level (non-compliance with the principles of well-being and excellence), and interpersonal level (non-compliance with respect, justice, etc.). Moreover, factors disrupting occupational mental health at the system level explored in job stress, a resilient culture, lack of managerial support, ingratitude, lack of reward-effort matching mechanism, and lack of resources.

## Introduction

Organizational occupational health as a key component in the sustainable development of systems commits organizations to ensure the physical and mental health of employees. The mental health of workers is a status in which a person realizes their abilities, copes with the normal pressures of life, increases their self-efficacy works more effectively, and helps their community. Increasing the psychological health of organizations leads to achieving organizational goals through the use of the potential talents of workers and available resources with better effectiveness [1]. A healthy work environment was defined as an environment in which the necessary factors were provided to ensure employees’ psychological health. The healthy work environment was recognized as a multidimensional concept that depended on various factors [2]. Muchisky defined nine factors that influenced on healthy work environment. The factors consist of the opportunity to control the environment, the opportunity to use skills, create external goals, diversity of the environment, environmental clarity, access to money, physical security, the opportunity to connect with others, and a valuable social status [3]. Healthy workplace standards were defined in six categories including skilled communication, true collaboration, effective decision-making, appropriate staffing, meaningful or reliable recognition, and authentic leadership [4]. Harmon updated occupational health standards for academic work and proposed self-care standards [2]. Day and Randell introduced an occupational health model with six elements for combining occupational health and well-being, including 1) Developing a culture of support, respect, and justice; 2) Creating opportunities for employee development and participation; 3) Creating and promoting psychological and physical security; and 4) Developing and promoting interpersonal relationships, 5) Ensuring the appropriateness and fairness of content and job characteristics, and 6) Encouraging a balance between personal and professional life [5].

The university, as an academic environment, requires providing the best conditions for employees’ well-being by observing occupational health standards. Academic work is a multi-dimensional activity that involves the physical, social, cognitive, and psychological dimensions. These activities are influenced by social, political, and cultural factors of the society [6]. Therefore, creating an environment that meets the criteria of organizational occupational health and well-being in universities is vital [6].

The main tasks of educators including various activities in the field of education, research, and executive management resulted in the acceleration of occupational burnout and lack of productivity in educators. Moreover, job-related functions in non-working hours, and organizational-managerial challenges, distorted the mental health, and quality of life of educators [7]. Lake and colleagues in a meta-analysis study revealed the positive associations between the work environment and the achieved outcomes of employers [8]. Occupational health is a complex issue that is influenced by cultural, social, and individual factors. Occupational health may be different in various environments [6]. Gray in a review study on workplace-based organizational interventions promoting mental health showed that high-income countries highlighted the importance of employee engagement in the intervention development and implementation process. Further studies were suggested on workplace mental health in low- and middle-income countries [9].

The present study was conducted in a developing country in which seven tasks, including education, research, executive duties, cultural development activities, personal development, healthcare services, and specialized activities outside the university were assigned to educators in the universities of medical sciences. The use of a sequential mixed-method design assists better understanding of the phenomenon by examining the current state using a quantitative method and explaining the associated factors by a qualitative study. The present study aimed to assess the status of workplace mental health from the perspective of educators and explore their experiences concerning occupational mental health factors at the Shahid Sadoughi University of Medical Sciences.

## Methods

This sequential mixed-method study was conducted in quantitative and qualitative phases. The sequential mixed method used the qualitative findings to help elaborate on or extend the quantitative results. An analysis of quantitative data in the first phase achieves extreme or outlier cases. Follow-up qualitative interviews with these outlier cases provide insight into the reasons for the diversity of the quantitative sample. In the sequential mixed-method design, quantitative methods were intended to achieve breadth of understanding of the phenomena, and qualitative methods were intended to achieve depth of understanding of the phenomena [10].

### Study setting

This study was conducted at Shahid Sadoughi University of Medical Sciences. The university includes six schools: medicine, nursing and midwifery, public health, paramedical sciences, pharmacy, and dentistry. All schools were represented in the present study. A total of 410 educators worked at the institution including (193 educators in basic sciences (47.7%) and 217 educators in clinical sciences (52.92)).

### Quantitative phase

#### Participants

Educators with at least two years of work experience contributed to this study. Educators were categorized according to their workplace into two categories clinical sciences who worked in hospitals (e.g. medicine, and allied medicine) and basic sciences who worked in schools (e.g., public health). The sample size was calculated to be 170 individuals, which considering z = 1.96, *σ*^2^ =194, d^2^ = 2.25 by formula $n=\frac{{\left({Z}_{1-\frac{\alpha }{2}}\right)}^2{\sigma}^2}{d^2}$, and added a 20% increase, it was estimated to be 203 participants. They contributed by a stratified random method. 205 educators (50%) participated in this phase (Table 1). In the study educators of various academic ranks including assistant professor, associate professor, and professor participated.

**Table 1:** Demographic characteristics of participants

		*N (%)*
**Gender**	Female	105 (51.21)
	Male	100 (48.78)
**Academic degree**	Assistance professor	100 (48.78)
	Associate professor	62 (30.24)
	Professor	43 (20.97)
**Faculty**	Clinical sciences	106 (51.70)
	Basic sciences	99 (48.29)
	*Mean (SD)*	
**Age**	42.87(7.30)	
**Working experiences**	9.8 (7.71)	

#### Measures

The Workplace Mental Health Questionnaire consists of 37 items in 9 categories including developing goals (*n* = 3), opportunity for control (*n* = 6), opportunity to use skills (*n* = 4), environmental diversity (*n* = 3), environmental clarity (*n* = 4), access to money (*n* = 3), physical security (*n* = 4), opportunity to contact others (*n* = 5) and valuable social status (*n* = 5). This questionnaire was developed and validated by Mehdad and colleagues. (Cronbach’s alpha = 0.94) [11, 12]. Each item is on a scale of 1–5, where 1 = never and 5 = always. The total scores of workplace mental health were categorized into three levels: low (the range scores of between 37 and 74), moderate (the range scores of between 74 and 148), and desirable (the range scores 148–185).

The anonymous questionnaire was distributed among the educators in different faculties. The participants completed the questionnaire by self-report. Data were collected from September 2022 to November 2022.

Quantitative data analysis: Data were analyzed by SPSS version 17 using descriptive statistics (frequency, median, interquartile range, mean and standard deviation).

### Qualitative phase

The experiences of the participants were explored using semi-structured interviews and were analyzed based on Graneheim and Lundman’s conventional content analysis approach [13]. In qualitative data collection, purposeful sampling was used so that participants who have experienced the phenomenon participated in the step [14]. The maximum variation sampling strategy was recommended to achieve the representativeness of participants with different experiences. This strategy is a common purposeful sampling used to identify and expand the range of variation or differences [10]. The maximum variation sampling assists in selecting those cases that are the most outstanding successes or failures related to some topic of interest. Such extreme successes or failures were to provide valuable information about the topic of interest [15]. In other words, the maximum variation sampling assists in explaining different aspects of the phenomenon by the range of participants who experienced it and identifying similarities and differences of the phenomenon of interest [10]. The strategy of maximum variation sampling was used to explore the experiences of educators who achieved the highest and lowest scores in Workplace Mental Health. This sampling helps to better understand the phenomenon.

In this study, 21 educators including 5 professors (23.80%), 10 associate professors (47.61%), and 6 assistant professors (28.57%) participated. They comprised 11 females (52.38) and 10 males (47.61%) with a mean (SD) age of 46 years [8] and work experience of 10 years [6].

#### Qualitative data collection

Before starting the interview, the study’s objectives were explained and, an informed consent form to participate in the research was obtained from the participants.

The time and location of interviews were arranged with the educators before the interview. Interviews were held in a quiet location in the interviewee’s office. In this phase, a semi-structured interview was conducted by a trained interviewer (a Ph.D. student). There was no relationship between the participants and the interviewer.

According to an interview guide, the interviews were started with the following questions: “What factors led you to decide to work as an educator at this university in your career? Is the faculty’s occupational mental health important at this university? How?, What factors did you experience that this university trouble you regarding physical and mental health to perform tasks as an educator?, Have you ever wanted to leave the profession or change your job?, What factors did you experience that caused a gap between the current status and desirable situation?”. As well, probing questions were used to explain educators’ experiences. Each interview lasted about 45 minutes on average. According to the participants’ informed consent, all interviews were recorded and transcribed completely.

#### Qualitative data analysis

Data were analyzed using the conventional content analysis approach introduced by Graneheim and Lundman. The interview text was listened to and reviewed. (at least three times). The process of data analysis was done in 6 stages, including 1. transcribing of interviews, 2. extracting the semantic unit and open codes, 3. summarizing and classifying the open code and selecting an appropriate label for them as a sub-category, 4. sorting sub-categories based on comparing similarities and differences in categories, 5. selecting a suitable title with the ability to cover the resulting categories, 6. combining categories explaining the theme and choosing the appropriate label [13]. So, data were categorized into three classes: open code, category, and theme [13]. The data were collected and analyzed in Persian and then translated into English for this paper. The results were translated and back-translated to English to ensure accuracy.

#### Rigor

Schwandt’s proposed criteria were used to ensure trustworthiness [16]. Various methods were used to the credibility of the data in the current study, including reflecting on the purpose of the study and the main research question, using an in-depth interview method with open-ended questions to gather information, and reflecting on semantic units to analyze long-term engagement with data. The extracted results were also reviewed by the participants. The transcripts of the interviews and analyses were returned to the participants and they were asked to review and confirm them (member check). The process of data analysis and extracted findings was also carefully examined by the research team members with experience in the field of qualitative research (peer check). Furthermore, two experts in qualitative studies audited and confirmed the data analysis process and extracted findings (expert check). Continuous comparisons between the content of categories and subcategories were used to observe semantic and structural coherence. In the present study, to facilitate the transferability of the data, a clear description of the background, sampling method, characteristics of the participants, the process of data collection, and the process of data analysis was reported.

### Ethical consideration

The present study was approved by the ethics committee of the National Agency Strategic Research in Medical Education. Tehran. Iran. (ID: IR.NASRME.REC.1401.017). In this study were considered the principles of confidentiality of the information and obtaining informed consent for an interview, recording the conversation, and having the right to withdraw from the research at any time.

## Results

### Quantitative phase

**Fig. 1:**
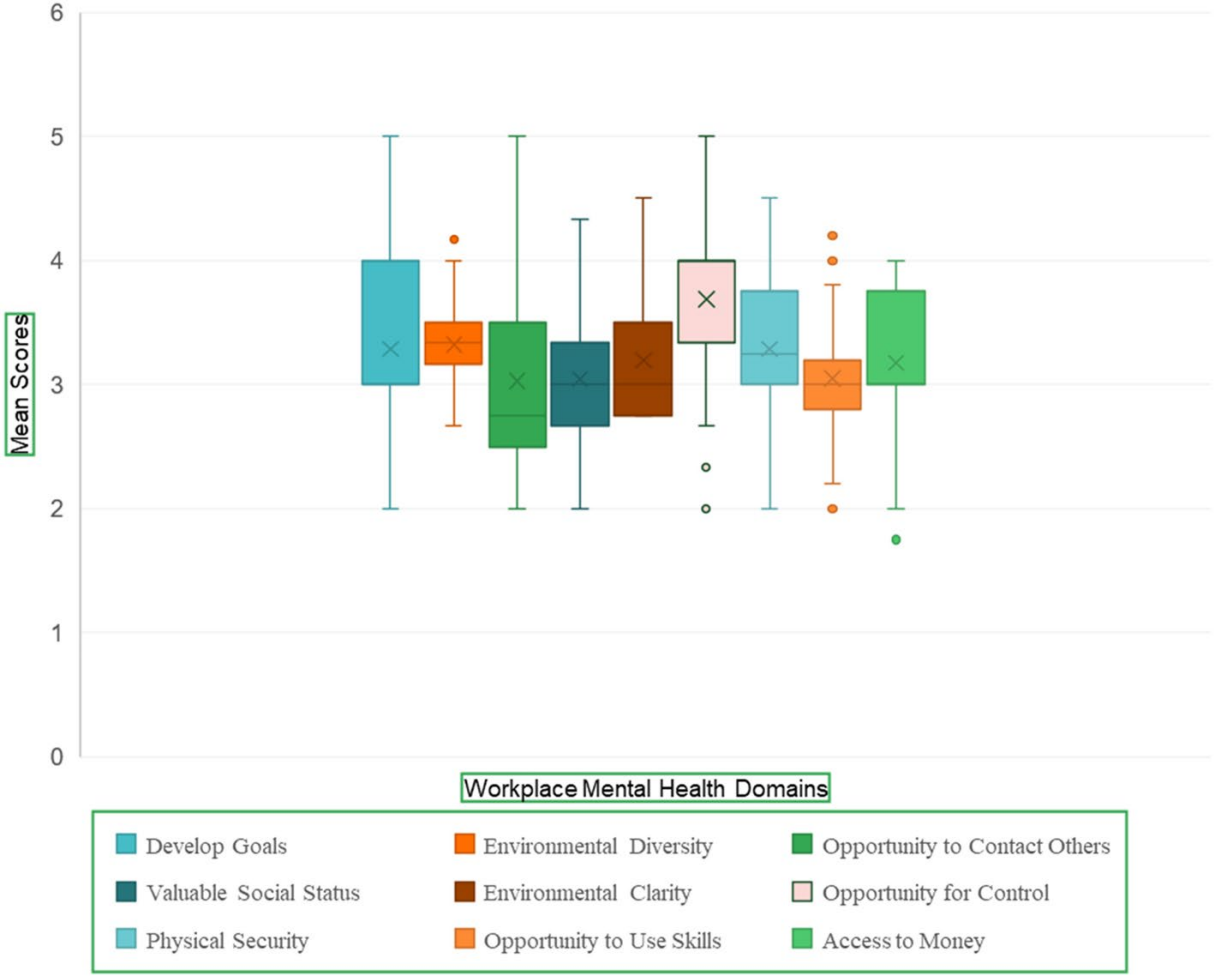
The educators’ perception in the workplace mental health domains

The results showed that the occupational mental health status of the educators was at a moderate level. (mean (± SD(= 115.87 (± 3.21). Figure 1 shows the educators’ perceptions in the domains of workplace mental health.

The highest and lowest scores of the median were reported in the domains of “opportunity for control” (median = 4) and “opportunity to contact others” (median = 2.75), respectively. The median as a middle value of a data set, means that 50% of data points had a value smaller or equal to the median and 50% of data points have a value higher or equal to the median.

### Qualitative phase

#### Contrast between preferences and disappointments in the development path

**Fig. 2:**
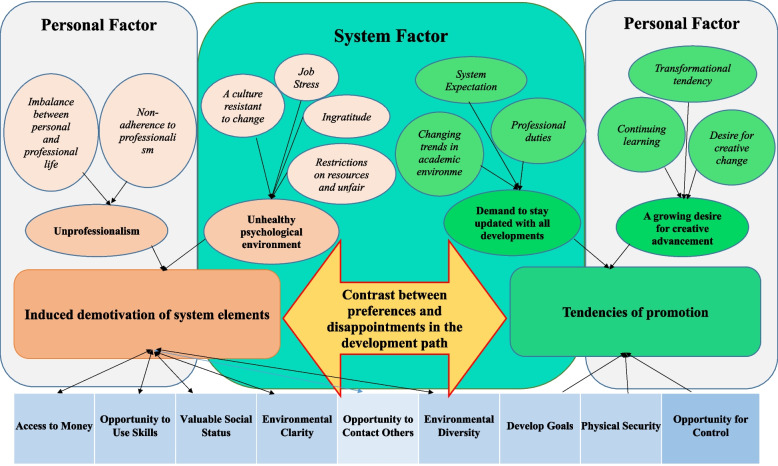
Mapping of quantitative and qualitative results within mental health occupational framework

The theme consists of two categories including “Induced demotivation of system elements” and “tendencies of promotion” (Fig. 2).

- A- Induced demotivation of system elements

In this category, the risk factors affecting occupational mental health were explained in two sub-categories “unhealthy psychological environment” and “unprofessionalism”.

- A 1- Unhealthy psychological environment

In this sub-category, job challenges such as job stress, and worries about the future were addressed. Furthermore, lack of resources with a negative impact on professional life and personal life, lack of support from managers, and ingratitude were discussed.

The sub-category addressed the sores of stress at the interpersonal and system levels. Negative competition, stressful relationships, individual perfectionism, responsiveness, low efforts of others, and fatigue caused by a high workload and stress at the interpersonal level. Moreover, the factors causing systemic stress such as job insecurity, various formal and informal duties, conflicting roles, and executive responsibilities were explained in this sub-category.

*I frequently encounter conflict in my role when I have multiple duties. I’m feeling extremely stressed. There is nothing I can do. I could not complete the tasks, which exacerbated my stress. (38-year-old female, assistant professor).*

*The stress of my job bothers me. I always have the stress of ensuring that my duties are done correctly. (40-year-old female, assistant professor).*

*Perfectionism causes me stress because I want to perform at my best in all duties and situations. I am always afraid that I won’t be able to do that effectively. (43-year-old woman, associate professor).*

The study found that an unhealthy work environment is caused by a culture of resistance to change and a lack of support from managers for change projects.

*The educators became obsessed with pleasure and self-indulgence. Anyone who takes this convenience away from them will be crushed by them. It’s their law. (48-year-old male, Professor).*

*Although I was an executive manager in the education department, I desired to depart. I had given up on taking on too much responsibility. My superiors were not supportive of me. They were unable to comprehend my efforts, and instead of appreciating me, they criticized my initiative. (53-year-old female, assistant professor).*

*Top managers considered the good results as their own; and if they were bad, they considered them as yours. I feel hopeless because of this. (47-year-old female, associate professor).*

Ingratitude, lack of appreciation, and supervisors’ perception of objectification were among the factors contributing to the unhealthy psychological environment.

*I was never given any encouragement, just reprimanded. (34-year-old male, assistant professor).*

*I worked to the best of my ability. My colleagues not only did not encourage me but also threw stones at me. I am feeling lonely, exhausted from the hard work, and running without any support. (35-year-old male, associate professor, executive manager).*

The sub-category addressed the economic concerns of educators including problems of resource restriction in professional and research activities, problems of livelihood and housing, and lack of welfare and worries about the future.

Regarding the limited financial resources for the development of professional activities and research budget, a participant stated:

*I wanted to do a new and product-oriented scholarship, but I did not get financial support. Many processes are slow, that is, it takes a long time to pay a budget and approve a thesis. The process was eroding. (45-year-old male, associate professor).*

The main sources of job stress were explained to be worrying about the future and the incompatibility of work and salary. A participant stated:

*Why are our salaries so low, even with the high expectations of educators in various fields? It hurts one’s mood. (35-year-old male, assistant professor).*

*My current concern is how to manage my life. These worries significantly diminish my ability to study, change, collaborate, and take responsibility. (65-year-old male, professor).*

- A 2- Unprofessionalism

Lack of adherence to the principles of professionalism, disrespect in interpersonal relationships, irresponsiveness of team members, discrimination, and imbalance of personal and professional life were among the unprofessional behaviors that turned the university environment into an unhealthy environment.

In regards to the lack of professionalism, sacrificing others for not taking responsibility and not fulfilling their responsibilities, an associate professor said:

*My dean made a sacrifice for me because he didn’t want to take on his responsibilities. Being a victim of the authorities’ game was something I did not want to experience and this was an awful feeling. (34-year-old female, assistant professor).*

Regarding discrimination, an educator said:

*Discomfort resulted from the unequal division of managerial positions. Sometimes one feels that they were a tool to help others gain an executive position. (43-year-old male, assistant professor).*

Obstruction was one of the unprofessional behaviors that a participant stated:

*Many individuals within the organization not only did not work but also prevented me from doing so. There was a tendency towards narrow-mindedness. This leads to someone experiencing attrition. (42-year-old male, associate professor).*

*After a break, my physical fatigue vanishes, but his mental fatigue persists for a long time. (32-year-old female, associate professor).*

The imbalance between personal and professional life was discussed as a common concern among educators, which is caused by an unhealthy workplace.

*The job of an educator is one of the most challenging and can cause physical and emotional fatigue. (42-year-old male, associate professor).*

*My family gave me negative feedback. Why were you so involved? I have a six-year-old child who is unable to comprehend, what can I say to him? He insists that I turn off my laptop. (34-year-old male, assistant professor)*

- B- Tendencies of promotion

The category addressed the system expectations and preferences of educators in an academic environment. Two subcategories were explored “a growing desire for creative advancement” and “demand to stay updated with all developments”.

- B 1- A growing desire for creative advancement

In this sub-category, the personal factors that impact the occupational health of educators were outlined. According to the educators’ perceptions, the dynamic environment at the university has enabled them to utilize their capabilities and creativity to optimize processes and enhance their achievements. They explained the opportunities and requirements for self-actualization in the academic environment, which led them to use their creativity to adapt to new trends in the academic process.

The pharmacy environment was dull and repetitive, which didn’t satisfy me. I am looking to enter an area that offers me diversity and opportunities for advancement. This opportunity was given to me by the university. (33-year-old female, assistant professor).

I am always interested in learning something new. I favored teaching new subjects in the teaching program. I take pleasure in learning and staying up-to-date. (48-year-old male, professor).

My mind is a catalyst for transformation. I am interested in making changes to my work, whether it involves research, care services, or education. Planning for improvement is something I enjoy. (49-year-old male, professor).

- B 2- Demand to stay updated with all developments

The requirement of universities to keep educators updated on specialized and developmental trends was discussed. The expectations have made participants experience the university as a dynamic environment. This dynamic environment has the potential to assist them in meeting their self-actualization needs and enhancing their performance.

*The university’s nature ensures that I am always up-to-date with the latest information. Staying up-to-date with the latest fashion trends is important to me, and it motivates me to persevere through hardships. (34-year-old female, assistant professor).*

*The university affords me the opportunity to plan and implement novel and innovative approaches to enhance processes in the community. (38-year-old male, associate* professor).

*Being static is something I don’t enjoy at all. I have a strong belief that I need to make progress in everything I do while working at the university. I must keep moving forward. (30-year-old female, assistant professor).*

## Discussion

Observance of occupational health requirements in universities was recognized as a key factor in sustainable development and organizational productivity. The current results showed that the occupational mental health status of educators was moderate. The highest and lowest scores of the median were reported in the domains of “opportunity for control” and “opportunity to contact others”, respectively. The experiences of educators were explored in the theme of “contrast between preferences and disappointments in the development path”.

The results indicated that the psychological health of educators was influenced by two factors, including the personal and system tendencies to progress and the perceived challenges of educators in the process of advancement. They believed that the nature of the university and its necessities require efforts to keep educators updated in various fields. The requirements were in line with educators’ interests in personal and professional development. In the developmental path, educators experienced demotivation factors in the university. These created a conflict and duality between preferences and requirements and expanded a demotivation climate in the university. These perceived factors have reduced the psychological and mental health of educators to an average level.

The current results indicated tendencies of promotion as internal motivators and external initiators experienced as a positive factor of occupational mental health among educators. The participants acknowledged their interest and tendencies to the promotion of personal and professional development as an intrinsic factor. The educators’ interest in continuous learning, creative problem solving, and dealing with scientific challenges motivated them and enhanced their satisfaction. In addition, the expectation of the academic environment for responding to changing trends is explored as an external initiator factor. The consistency of the dynamic nature of academic environments, and educators’ tendencies of promotion in different fields were explored as a positive factor of the occupation mental health of educators in the university. In line with our results, Wan-Shuai and colleagues indicated educators’ development is a systematic process which influenced by external behavior and internal states of educators. Educators were encouraged to optimize their practice according to their values and external tasks in the institute [17]. Similarly, Zhang and colleagues explained that teachers’ motivation for learning was associated with self-efficacy, beliefs about learning, and emotional pressure as a personal factor. Moreover, leadership and organizational principles as system factors to increase their motivation and commitment to participate in professional learning activities were explained [18]. In contrast to the current results, Alves and colleagues indicated encountering new methods and trends in education and research distorted the mental, health, and educational performance of educators [7]. The results may be influenced by the organizational expectations, and preferences of educators in the universities [7, 9]. Although different academic rank classes participated in this study, the proportion of assistant professors was higher in the investigated university than in other classes, which may affect the results.

Communication, and interaction of workers at different levels of the system were considered as main elements of organizational occupational health [2]. The lowest median scores in the domain of “opportunity to contact others” were reported from the perspective of educators. The domain assessed cooperation with other colleagues, the conditions for using the experience of colleagues in doing work, and the value of collective and teamwork in an organization. The results showed that more than half of the educators reported a poor situation (below moderate level) in this domain. In line with this quantitative finding, the category of unprofessionalism was explored as a main risk factor for psychological health. Disrespect in interpersonal relationships, the irresponsiveness of team members, and discrimination harmed the opportunity to contact others. Distorting interpersonal and inter-professional interactions resulted in discouragement and demotivation of educators. These risk factors turned the university environment into an unhealthy environment. Cochran introduced effective and supportive communication, and professional development by teamwork in the organization as effective factors which if not observed, cause an unhealthy work environment [19]. Similarly, in a meta-analysis, job-related factors such as workload and ineffective communication at work were identified as key factors affecting participants’ motivation and burnout [20].

Assistant professors as young educators believed that they experienced unprofessional behaviors such as disrespect in interactions, discriminatory behaviors in receiving privileges, being forced to perform more difficult tasks, and being abused by junior professors and top managers. These led to the elimination of the motivation of senior educators. In line with the present study, Fitchett and colleagues showed the association between first-year teachers’ risk for stress and professional preparation. They indicated teacher education and support programming associated with the risk for stress classification that teachers experienced [21]. Kelly and colleagues suggested the support of personal resilience-building activities, which require planning especially with younger members. Because young members of the system were more prone to burnout [22]. Furthermore, Chiou-Fen recommended supporting employers through planning career development and teamwork opportunities [23].

The ‘environmental diversity’ domain addressed the variety of tasks and dynamic environment in the institute. The educators’ scores in this domain were reported at higher than the median. The high diversity of the tasks for educators was explored as a risk factor for a healthy workplace in the qualitative results. The participants believed that multiple tasks, workloads, and conflicting roles and tasks, caused job stress among them. They believed not to be able to manage various tasks and high expectations of the institute. These were explored as the key factors of elimination of their psychological health. Likewise, Faisal and colleagues identified workplace factors comprising work overload, role conflict, and management ineffectiveness as sources of stress for university teachers [24]. A review study showed that job expectations (such as workload, job characteristics, and conflict of values), and lack of resources (including social support and rewards) have a significant effect on burnout [25]. A self-care is proposed as a new standard in the workplace occupational health framework. Educators were expected to actively participate in self-care activities to create a healthy job and a favorable learning environment [2]. The participants believed that due to the high workload and various tasks, they had to do work activities during non-working hours and on weekends. They considered the restriction of time, family dissatisfaction, mental fatigue, and lack of enough rest as factors accelerating their unhealthy mental status. The results of studies confirmed the negative relationship between physical and mental well-being with burnout, poor performance in the team and systems, and reduced productivity [20, 26]. The results of Alves and colleagues showed that educators who felt tired before starting work reported lower quality of life, and those who needed more time to rest after work reported less satisfaction. These results can be due to non-compliance with excellence principles for creating a balance between personal and professional life [27].

Organizations affect occupational mental health through changes in work environments, level of organizational support, and a safe psychological atmosphere [5]. The participants believed that in this university, there was resistance to changes atmosphere in the system. Then, any program that leads to change and disrupts the convenience of educators was not accepted. This leads to unprofessional behaviors such as slander, and disrespect to the executive authorities of change programs. The lack of support from supervisors and coworkers in the change process was explored as a main risk factor for the mental health of educators. Likewise, Sabagh acknowledged stress and lack of support have negative effects on faculty’s commitment and performance and endangers their mental and physical health [25]. López-Cabarcos identified support from their supervisors or coworkers as the main factor in a healthy work environment that improved employees’ job performance [28].

In this study, ingratitude was mainly experienced by educators who had executive responsibilities. The participants believed that the incompatibility of individual characteristics such as transformational preferences with the university culture as a restriction to change were among the factors disrupting the occupational mental health of the educators. Lack of support for change programs, ignoring the efforts of educators, and creating challenges for the change team was classified in the sub-category. Gratitude when job stress is high and burnout is considered a concern in the organization has a positive impact on the professional life of the employees [22, 29]. Faisal and colleagues showed recognition as appreciation, gratitude, and admiration for outstanding performance explored as a key factor in the health work environment. They acknowledged appreciation and gratitude of teachers led to the feeling that they were valued by the organization [24]. Afzal acknowledged admiration, constructive criticism, and positive feedback enhanced the engagement and workplace flourishing of faculty members [30]. The establishment appreciation mechanism is recommended for educators.

The score of the domain of access to money and financial resources was reported at the intermediate level by educators. In the qualitative phase, economic problems, and restriction of resources was explored as a negative factor. These factors harmed the personal and professional lives of educators. This study was conducted in a developing country where there was economic instability. The educators believed that although access to money was mediocre, economic instability made them worried about the future. Recently, the enactment of new tax laws and the setting of salary limits for educators has also caused serious concern among educators. These concerns and the incompatibility of work and salary resulted in their demotivation. Similar to the present study, the concern for housing, income, and the management of future financial problems was troubling for academic members and was referred to as a main factor of job stress in Arian’s study [31]. In line with our results, Alves and colleagues addressed facilities such as a house, sufficient income, and life insurance as the minimum necessary facilities that affected educators’ mental health [7].

The Day’s occupational model explained justice as the main component of a healthy workplace. The present results showed a lack of appropriate evaluation, and feedback, and injustice reward systems had led to demotivation, abandonment of responsibility, and reduced effort of educators [32]. Perceiving injustice in the distribution of resources and privileges and interpersonal interactions was positively related to mental health problems and employees’ stress disorders [33]. Similar to the present study, the effort-reward imbalance was strongly associated with an increased risk of mental disorders such as depression, anxiety, and organizational disorders in the workplace [32]. Faisal’s results showed the disparity of rewards, missing supportive feedback, the culture of favoritism/biases, and low salary as the main challenges of healthy work from the perception of faculty members [24].

The development of evaluation systems and feedback-reward mechanisms is recommended for educators who have multiple responsibilities (teaching, research, and executive management) in medical sciences universities. Gorgenyi-Hegyes and colleagues suggested the development of employees, and wellness were planned as occupational health promotion activities [34]. In addition, the implementation of supportive policies for occupational health, and empowerment programs in the field of psychological and self-care is suggested to plan in the university.

### Limitations

A small sample size in the quantitative phase and exploration of educators’ experiences in one university were limitations of this study. The data in the quantitative phase were collected by self-report that may some participants tend to respond in the middle of the scale. A main concern in the phase was social desirability bias, when participants were susceptible to give answers that they consider to be the most socially acceptable one. Moreover, the study was conducted in a developing country, and the generalizability of the findings to other settings was a restriction of the study.

## Conclusion

University, as a dynamic environment, needs to pay attention to the components of occupational mental health for the best productivity of the educators. The results showed that the occupational mental health of educators was moderate. Factors causing unprofessionalism and unhealthy psychological work environments at both interpersonal and systemic levels harmed the occupational mental health of educators in the investigated university. The gap between the current situation and the desired state of occupational mental health may be due to cultural challenges of non-adherence to professionalism at the individual level (non-compliance with the principles of well-being and excellence) and interpersonal level (non-compliance with respect, to justice, etc.). Moreover, factors that affect occupational mental health at the system level include job stress, resilience to change culture, lack of managerial support, reward-effort matching mechanism, ingratitude, and resource restriction. It is recommended to pay attention to the elements of occupational health, by developing support-incentive programs and creating a culture of staff support in the universities. The tendency of educators for promotion was explored as a positive factor in the mental health of educators in the academic environment. A growing desire for creative advancement among educators as a personal factor and demand to stay up-to-date with all developments as a system factor were explained the positive experiences of educators in the university.

## Data Availability

The datasets generated and/or analyzed during the current study are not publicly available due to the confidentiality of the data of participants but are available from the corresponding author at reasonable request.
